# Improving Alzheimer’s Disease Prediction with Different Machine Learning Approaches and Feature Selection Techniques

**DOI:** 10.3390/diagnostics14192237

**Published:** 2024-10-07

**Authors:** Hala Alshamlan, Arwa Alwassel, Atheer Banafa, Layan Alsaleem

**Affiliations:** Department of Information Technology, College of Computer and Information Sciences, King Saud University, P.O. Box 51178, Riyadh 11543, Saudi Arabia; 442202188@student.ksu.edu.sa (A.A.); 442203479@student.ksu.edu.sa (A.B.); 442201879@student.ksu.edu.sa (L.A.)

**Keywords:** machine learning, OASIS, prediction, Alzheimer’s disease (AD), feature selection

## Abstract

Machine learning (ML) has increasingly been utilized in healthcare to facilitate disease diagnosis and prediction. This study focuses on predicting Alzheimer’s disease (AD) through the development and comparison of ML models using Support Vector Machine (SVM), Random Forest (RF), and Logistic Regression (LR) algorithms. Additionally, feature selection techniques including Minimum Redundancy Maximum Relevance (mRMR) and Mutual Information (MI) were employed to enhance the model performance. The research methodology involved training and testing these models on the OASIS-2 dataset, evaluating their predictive accuracies. Notably, LR combined with mRMR achieved the highest accuracy of 99.08% in predicting AD. These findings underscore the efficacy of ML algorithms in AD prediction and highlight the utility of the feature selection methods in improving the model performance. This study contributes to the ongoing efforts to leverage ML for more accurate disease prognosis and underscores the potential of these techniques in advancing clinical decision-making.

## 1. Introduction

Dementia, a cognitive impairment characterized by deficits in thinking, remembering, and reasoning abilities, as well as behavioral impairments, hinders an individual’s daily functioning and activities. Its severity varies from an incipient stage that marginally affects functioning to an advanced stage where the individual becomes entirely reliant on others for the basic activities of daily living. Neurological alterations may commence a decade or more before the manifestation of clinical symptoms in Alzheimer’s disease. During this early stage, a series of toxic changes occur in the brain, including the abnormal aggregation of proteins leading to the formation of amyloid plaques and tau tangles. Healthy neurons cease to function properly, lose connections with their neighboring neurons, and ultimately degenerate. Numerous other intricate modifications in the brain are believed to contribute to the pathology of Alzheimer’s disease [1].

Alzheimer’s disease (AD) is the most common type of dementia. It is a progressive disease beginning with mild memory loss and possibly leading to the loss of the ability to carry on a conversation and respond to the environment. However, early accurate diagnosis can affect the patient’s outcomes and slow the decline in memory. More focus has been shifted towards risk reduction, leading to new discoveries and solutions. The research indicates that individuals with healthy lifestyle habits are less likely to develop dementia. Also, behaviors proven to prevent diseases such as cancer and diabetes may also lower the risk of cognitive decline [2].

As a division of artificial intelligence (AI) and computer science, machine learning (ML) involves using data and algorithms to enable AI to replicate human learning behaviors [3]. The increased adoption of machine learning in healthcare has created new possibilities for disease diagnosis and treatment [4]. Relevant data and patterns can be identified by applying machine learning to patient datasets stored in electronic health records [5]. Machine learning algorithms have been playing a vital role in solving complex, highly nonlinear classification and prediction problems [6].

There is a pressing need for more research on the causes of Alzheimer’s disease (AD), as well as on the early diagnosis and treatment strategies. Our study focuses on using machine learning to aid in the early diagnosis of AD and to evaluate the effectiveness of various algorithms. Given the increasing rates of AD cases, effective early detection strategies are needed. By employing machine learning, we aim to enhance early detection.

The objective of this study is to investigate various machine learning (ML) models such as Support Vector Machine, Logistic Regression, and Random Forest, along with different feature selection methods such as Minimum Redundancy Maximum Relevancy and Mutual Information, aimed at evaluating the effectiveness of each model and eventually advancing their application in healthcare through supervised learning methodologies. By the end of this study, we develop a total of 72 different models with accuracies ranging from 91.43% to 99.08%. The structure of this paper is organized as follows: Section 2 provides the background on the techniques utilized in this study, including Support Vector Machine, Random Forest, and Logistic Regression, along with feature selection methods such as Minimum Redundancy Maximum Relevance and Mutual Information. Section 3 presents a literature review of the relevant research conducted on machine learning models for predicting dementia and Alzheimer’s disease. Section 4 outlines the materials and methods employed in this study, detailing the data collection, preparation, and analysis processes. Section 5 discusses the results obtained from the various machine learning models, including a comparison of their performances. Finally, Section 6 concludes the paper with a discussion regarding the findings and their implications for future research.

## 2. Background

The techniques used in this study, such as SVM, Random Forest, Logistic Regression, Mutual Information, and mRMR, will be discussed in this section.

Support Vector Machine (SVM) is a supervised learning algorithm in machine learning, effective for tasks like classification and regression. It excels particularly in binary classification, where it categorizes data into two groups [7].

Logistic Regression (LR) is a supervised machine learning algorithm utilized for classification tasks, where the objective is to predict the probability of an instance belonging to a particular class. It is well-suited for binary classification problems [8]. The Random Forest (RF) algorithm consists of an ensemble of Decision Trees. Each tree in the ensemble is constructed using a bootstrap sample, drawn with replacement from the training set. This algorithm is known for its robustness and ability to handle complex datasets [9].

mRMR, which stands for ‘Minimum Redundancy Maximum Relevancy’, is a feature selection method that aims to select the most relevant attributes from the dataset while minimizing redundancy among them [10].

The relevance of each feature is evaluated based on its correlation and relevance with the target variable and redundancy among other features. Features with high relevancy and low redundancy are prioritized and selected [10].

mRMR is usually formulated as follows [11]:

(1) $${\mathrm{score}}_{i}\left(f\right)=\frac{F(f,\mathrm{target})}{\sum_{s\in \mathrm{features}\;\mathrm{selected}\;\mathrm{until}\;i-1}\frac{\left|\mathrm{corr}(f,s)\right|}{i-1}}$$

The correlation coefficient quantifies the strength of the linear relationship between two variables, ranging from −1 to 1. −1 indicates a perfect inverse correlation (when a value in one series increases, another value decreases), 1 indicates a perfect positive correlation (when a value in one series increases, another value rises), and 0 denotes no linear relationship [12].

The Pearson correlation coefficient, denoted as r, is calculated using the following formula [13]:

(2) $$r=\frac{n(\sum xy)-(\sum x)(\sum y)}{\sqrt{\left[n\sum x^{2}-{\left(\sum x\right)}^{2}\right]\left[n\sum y^{2}-{\left(\sum y\right)}^{2}\right]}}$$

Mutual Information (MI) estimates relationships between fixed categories in classification problems and continuous variables in regression. It is based on the entropy of the variables involved. MI is a measure of how much two random variables depend on each other. It is a non-negative value that equals zero when the variables are independent, meaning they do not affect each other. Higher MI values indicate stronger dependencies.

The MI between two random variables X and Y is as follows:

(3) I(X;Y)=H(X)−H(X|Y)

In this equation, *I(X;Y)* represents the MI for X and Y, H(X) denotes the entropy for X, and H(X | Y) is the conditional entropy of X given Y. The result is measured in bits, with values ranging from zero to one [14].

## 3. Literature Review

In this section, we discuss and review research studies conducted between 2019 and 2023 on the subject of using ML models in predicting dementia and Alzheimer’s disease (AD). These studies utilize the OASIS longitudinal dataset, known as OASIS-2 [15]. The dataset contains 15 columns and 373 rows.

Using both the OASIS-1 and OASIS-2 datasets, Saratxaga et al. [16] proposed the use of deep learning (DL) techniques, as opposed to the traditional image processing and classification methods, in regard to MRI analysis. The study aims to calculate the accuracy and balanced accuracy and compare the results to previous works. Two approaches are used: 2D slice-level and 3D subject-level. Various architectures were used on the OASIS-2 subset, including BrainNet2D, BrainNet3D, and ResNet18. Batch Normalization and Cyclical Learning Rate (CLR) were used to increase the performance.

As for the results, BrainNet2D using CLR triangular achieved an accuracy and baseline accuracy of 92%, and BrainNet3D using Batch Normalization had an accuracy and baseline accuracy of 84%. ResNet18 using CLR triangular and min–max scaling achieved the highest accuracy and baseline accuracy of 93%.

Battineni et al. [17] used Support Vector Machines (SVMs) and achieved 68.75% accuracy and 64.18% precision. These findings substantiate that superior predictive performance in dementia prognosis is obtained with low gamma (1.0 $\times 10^{-4}$) and high regularized (C = 100) values. Kavitha et al. [18] employed various classifiers, including the Random Forest (RF) technique, with median imputation utilized for addressing the missing values in SES. The RF classifier yielded an accuracy of 86.92%.

Baglat et al. [19], in addition to using both SVM and RF, used Logistic Regression (LR) and Decision Trees (DTs) techniques, with the RF performance achieving the best results. The RF classifier achieved an accuracy of 86.8%. AdaBoost, a boosting algorithm, was implemented to increase the predictive accuracy.

Basheer et al. [20] proposed a different approach, introducing a Modified Capsule Network model, M-CapNet. To increase the performance and accuracy of the model, several optimization techniques were applied. The model achieved 92.39% accuracy.

Dhakal et al. [21] applied various additional ML techniques, including K-Nearest Neighbor (KNN). These techniques were used on features selected using Least Absolute Shrinkage and Selection Operator (LASSO) and the full set of features. Upon comparing the metrics, the SVM model achieved the highest accuracy of 96.77%.

Overall, the reviewed works have shown promising advancements in the utilization of ML models for predicting dementia and Alzheimer’s disease. These studies have explored various ML techniques, including deep learning (DL) approaches like BrainNet2D, BrainNet3D, and ResNet18, as well as Support Vector Machines (SVMs), Random Forest (RF), Logistic Regression (LR), Decision Trees (DTs), and Modified Capsule Network (M-CapNet). In Table 1 and Table 2, we present the comparison between all the methods and the advantages and disadvantages of each study, respectively.

## 4. Materials and Methods

In this section, we will discuss the stages we went through, from collecting, preparing, and processing data to choosing features and models to arrive at our proposed model, which achieves the highest percentage of accuracy.

### 4.1. Data Collection and Preparation

In this study, we used the OASIS-2 dataset [15], which consists of a longitudinal collection of 150 people aged 60–96 years. Each subject was scanned on two or more visits, separated by at least one year, for a total of 373 imaging sessions. For each subject, 3 or 4 individual T1-weighted MRI scans were included in individual scanning session [22].

All participants in this dataset are right-handed and include both men and women; 72 people were classified as non-demented throughout the study, while another 64 were described as having dementia upon their initial visits, which remained so for subsequent scans, including 51 individuals with mild to moderate Alzheimer’s disease [22]. Another 14 subjects were characterized as non-demented at the time of their initial visit and were subsequently characterized as demented at a later visit [22].

This dataset includes 373 instances and includes 10 unique features shown in Table 3 below.

### 4.2. Data Analysis

**Figure 1:**
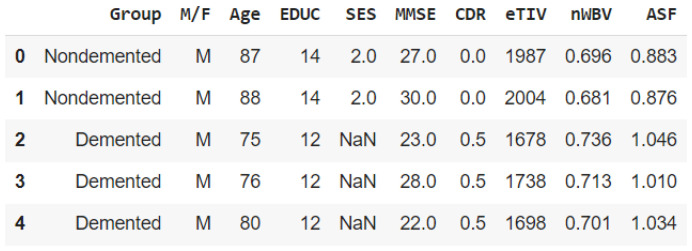
The result of first 5 rows.

**Figure 2:**
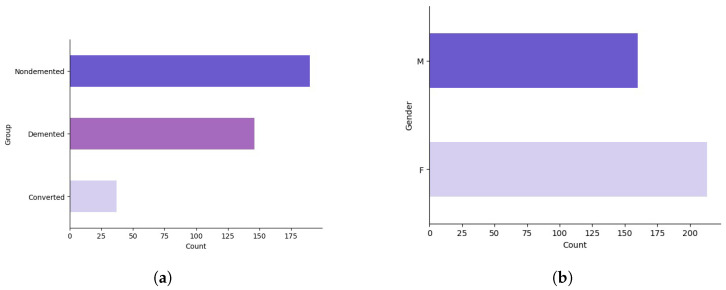
Descriptive statistics of the participant demographics within the dataset. (**a**) The distribution of group classifications. (**b**) The gender representation.

Initially, we employed basic analytical techniques to delineate the properties of each feature, thereby enhancing our understanding of the dataset’s characteristics. This foundational analysis is illustrated in Figure 1 and Figure 2a,b.

All features in the dataset showed numeric properties except “gender” (classified as “M/F”) and “Group” (serves as a class label).

#### 4.2.1. Data Transformation

As we can see in Table 4, the “Group” feature, serving as a class label, is divided into three categories. Knowing that the converted column does not contribute to the classification process in the integrated machine learning model, we decided to combine this category with the demented category to achieve a balanced dataset, so the dataset will now contain 190 records. These are classified as non-demented and 183 records are classified as demented.

#### 4.2.2. Encoding

As we can see in our dataset, we have two columns (group and M/F) that we need to use label encoding to ensure compatibility; the result is shown in Table 5 below:

#### 4.2.3. Missing Values

**Figure 3:**
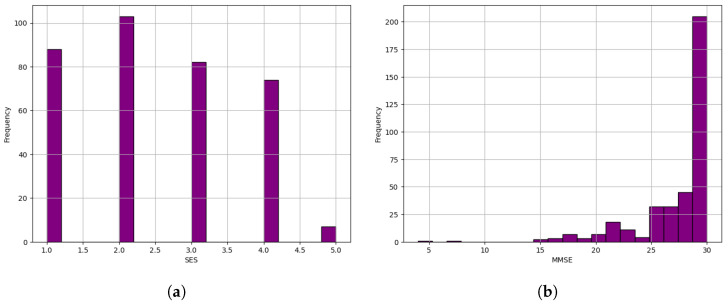
Histograms illustrating the distribution of socioeconomic status. (**a**) SES scores among participants. (**b**) MMSE scores among participants.

When we examined the dataset, we noticed 19 missing values in the “SES” column and two missing values in the “MMSE” column. To choose the appropriate method for dealing with the missing values, we analyzed both columns, considering replacing the missing values with either the mean or the median, as shown in Figure 3.

Given the non-normal distribution observed in both columns, we chose the median as the most appropriate method for dealing with missing values. This choice is supported by the resilience of the median to the disproportionate effect of the outliers compared to the mean, making it a more robust measure of central tendency.

#### 4.2.4. Outlier Analysis

**Figure 4:**
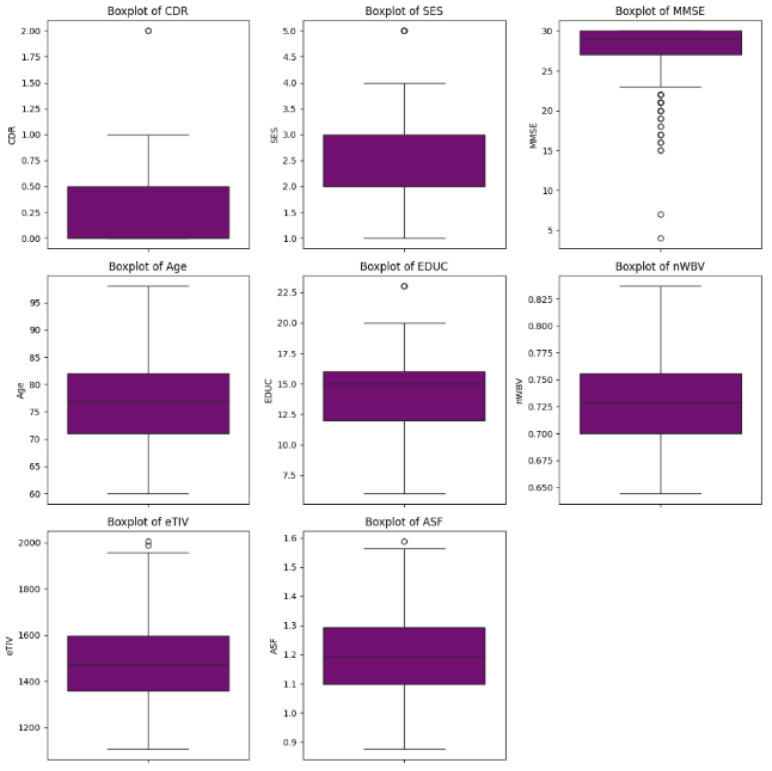
Boxplots representing the distribution and variability of numeric attributes within the dataset, facilitating the identification of potential outliers.

**Figure 5:**
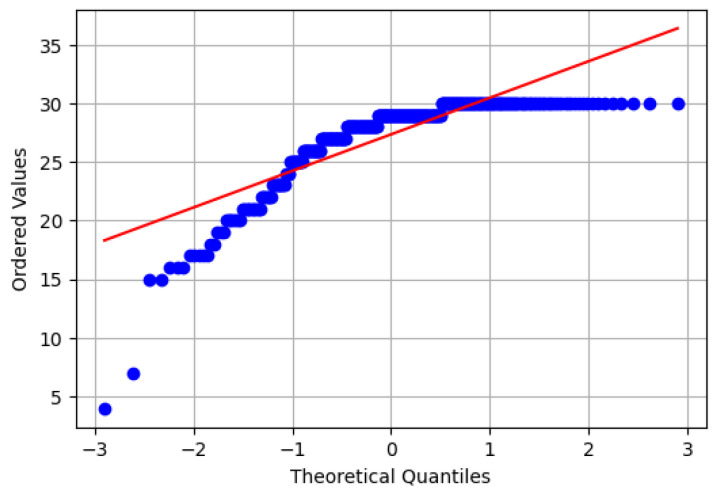
Probability plot depicting the distribution of MMSE scores, highlighting the assessment of cognitive function among participants.

Before determining the appropriate method for dealing with outliers, it is necessary to analyze them, confirm their nature, and determine whether they are real outliers or those arising from human error. In light of this, we analyzed them to identify the nature of the outliers, as shown in Figure 4; it is clear that some bars show outliers. However, our attention was particularly drawn to outliers in the MMSE column, prompting us to further investigate them, as shown in Figure 5.

As shown in Figure 5, the highest value of the MMSE score is 30. A score of 25 or higher is classified as natural. If the score is less than 24, the score usually indicates possible cognitive impairment [23] and is an abnormal result. Therefore, it can be concluded that the outliers observed in the MMSE column are inherent in the distribution of the data and do not arise from human error. As a result, the decision was made to retain these outliers within the dataset.

### 4.3. Feature Selection

In the feature selection process, we used three methodologies for feature selection: mRMR (Minimum Redundancy Maximum Relevance), correlation coefficient analysis, and Mutual Information methodology. Our intent was to select a subset of five features as an initial step in the feature selection process.

**Figure 6:**
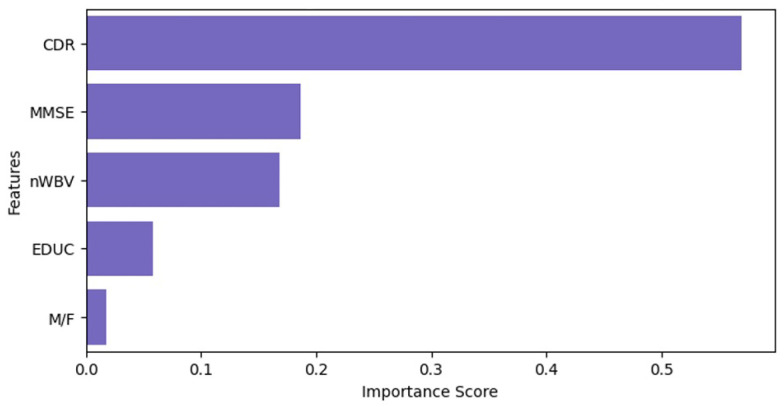
Visualization of feature selection results using mRMR.

**Figure 7:**
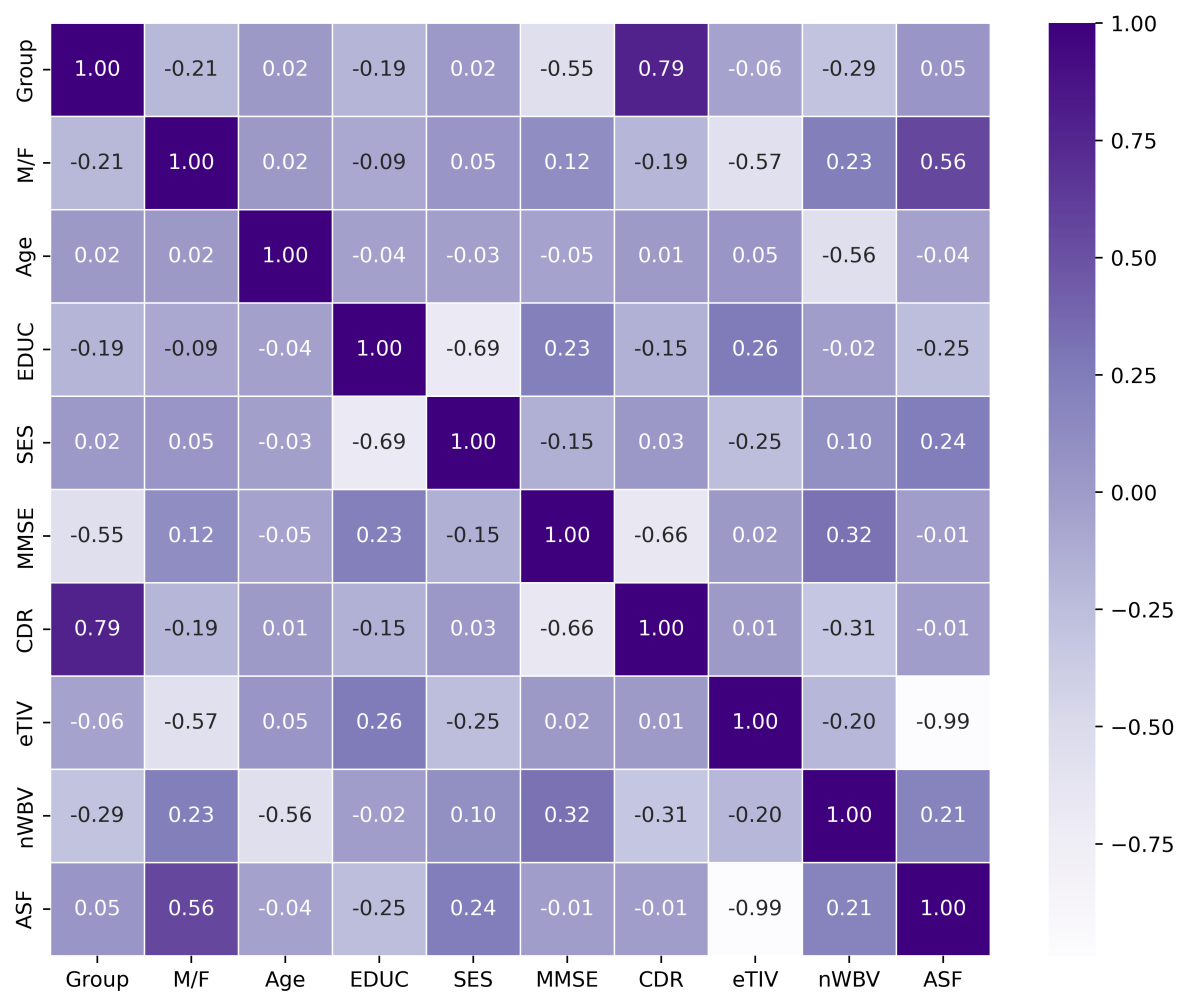
Heat map of correlation coefficient feature selection.

The results derived from the application of mRMR and correlation coefficient methodologies are shown in Figure 6 and Figure 7.

First, we analyzed mRMR and correlation coefficient and found that both methodologies yielded identical results, namely CDR, MMSE, nWBV, EDUC, and M/F. However, these results were not sufficient to satisfy our curiosity, so we decided to use another approach: Mutual Information.

**Figure 8:**
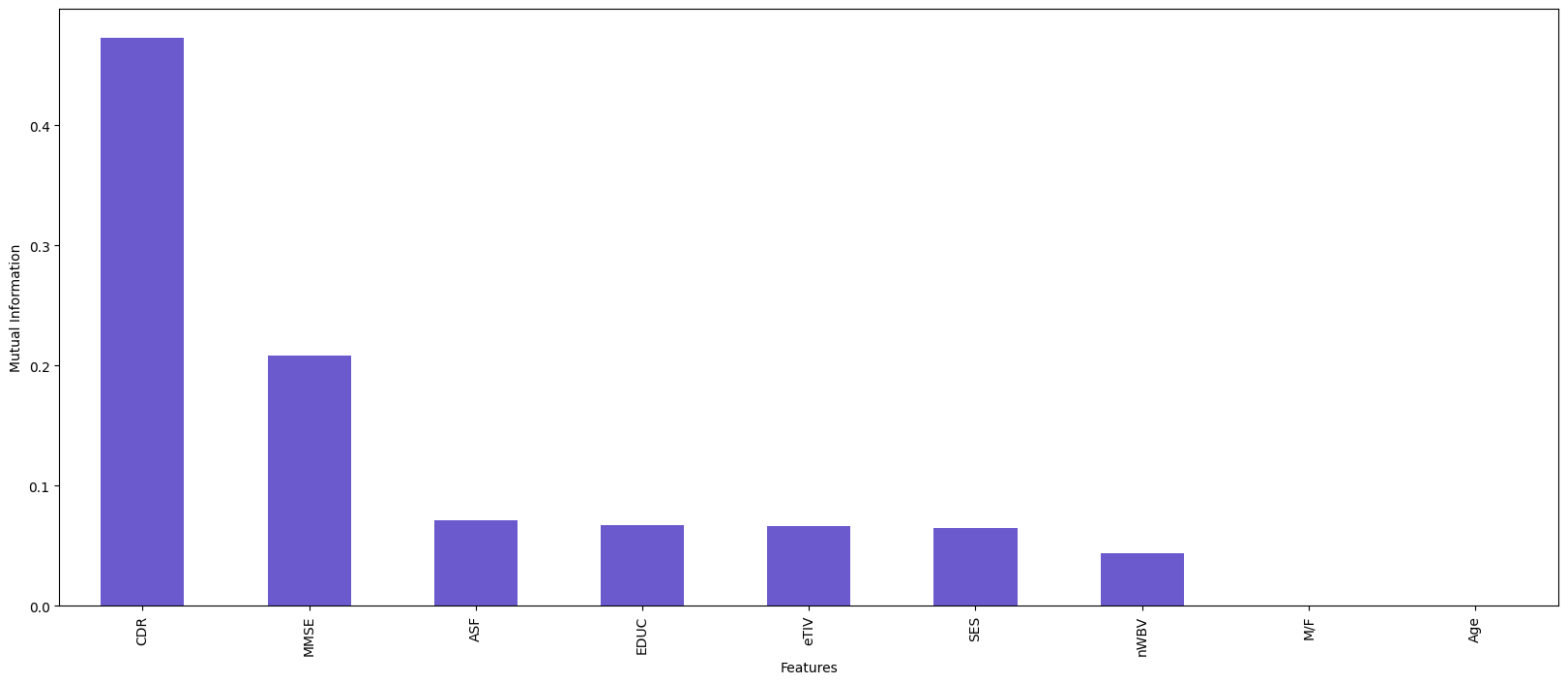
Mutual Information feature selection.

The result after applying Mutual Information is shown in Figure 8.

The result is a little different from the previous methodologies; the first five columns identified through the Mutual Information methodology are CDR, MMSE, EDUC, eTIV, and ASF.

Since mRMR and correlation coefficient yielded identical results, we decided to use mRMR and Mutual Information in our model; the outcomes of feature selection methodologies are shown in Table 6 below.

### 4.4. Classifier Models

We used three classification methods: Support Vector Machine (SVM), Random Forest, and Logistic Regression models. Each model was trained without feature selection, and with mRMR selection and Mutual Information feature selection methodologies. We also tried the cross-validation method, which we will discuss in the Section 5.

## 5. Results and Analysis

This section presents the findings from the various machine learning models employed in this study, focusing on their predictive performance. Both the hold-out and cross-validation methods were utilized to evaluate the model accuracy, highlighting the effectiveness of the selected algorithms and feature selection techniques in predicting Alzheimer’s disease.

### 5.1. Hold-Out Method

In this subsection, we detail the results obtained through the hold-out method, which involves dividing the dataset into training and testing subsets to assess the models’ performance. The following results focus on the Support Vector Machine (SVM), Logistic Regression, and Random Forest models, showcasing the predictive capabilities under various feature selection techniques.

#### 5.1.1. SVM

**Figure 9:**
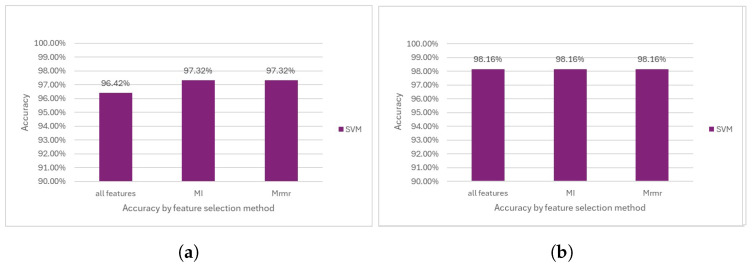
Performance comparison of the SVM model. (**a**) Accuracy before outlier removal. (**b**) Accuracy after outlier removal.

The Support Vector Machine (SVM) model consistently achieved an accuracy of 97.32% when utilizing both the mRMR and MI feature selection methodologies. In contrast, when all the features were considered, the model attained an accuracy of 96.42% (see Figure 9a). Notably, the performance of the SVM model improved significantly following the removal of the outliers, with accuracies reaching 98% across all the methodologies (see Figure 9b).

#### 5.1.2. Logistic Regression

**Figure 10:**
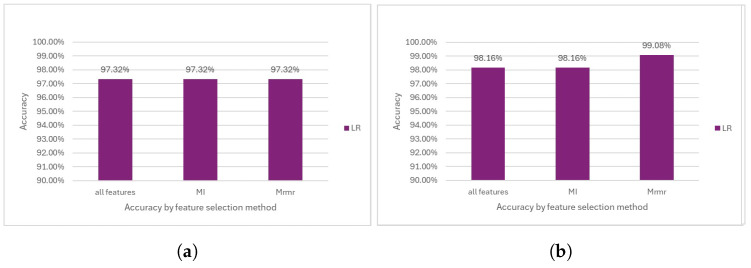
Performance comparison of the LR model. (**a**) Accuracy before outlier removal. (**b**) Accuracy after outlier removal.

Before the removal of the outliers, LR showed similar results to SVM despite their different methods, demonstrating the effectiveness of both models in achieving high accuracy (see Figure 10a).

The results from LR after removing the outliers displayed an overall improvement across all the methodologies, with accuracies ranging from 98% to 99%. This enhancement is evidenced by the highest accuracy achieved at 99% (see Figure 10b).

#### 5.1.3. Random Forest

**Figure 11:**
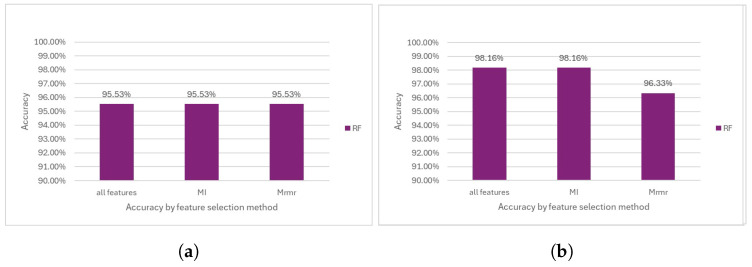
Performance comparison of the RF model. (**a**) Accuarcy before outlier removal. (**b**) Accuracy after outlier removal.

RF appears to perform slightly lower than SVM and LR. The highest accuracy achieved was recorded at 95.53% for all the feature selection methods; however, it is still lower compared to the accuracy achieved by the other two methodologies (see Figure 11a).

The outcomes derived from RF after removing the outliers exhibited notable enhancements, with accuracies ranging from nearly 96% to 98%, indicating significant advancements in performance (see Figure 11b).

### 5.2. Cross-Validation

To further evaluate the robustness of our models, we employed k-fold cross-validation, a technique that enhances the reliability of accuracy estimates by utilizing multiple subsets of the data. We tested various configurations of K in the k-fold cross-validation process, specifically using K = 10, K = 5, and Leave-One-Out (LOO) strategies.

**Figure 12:**
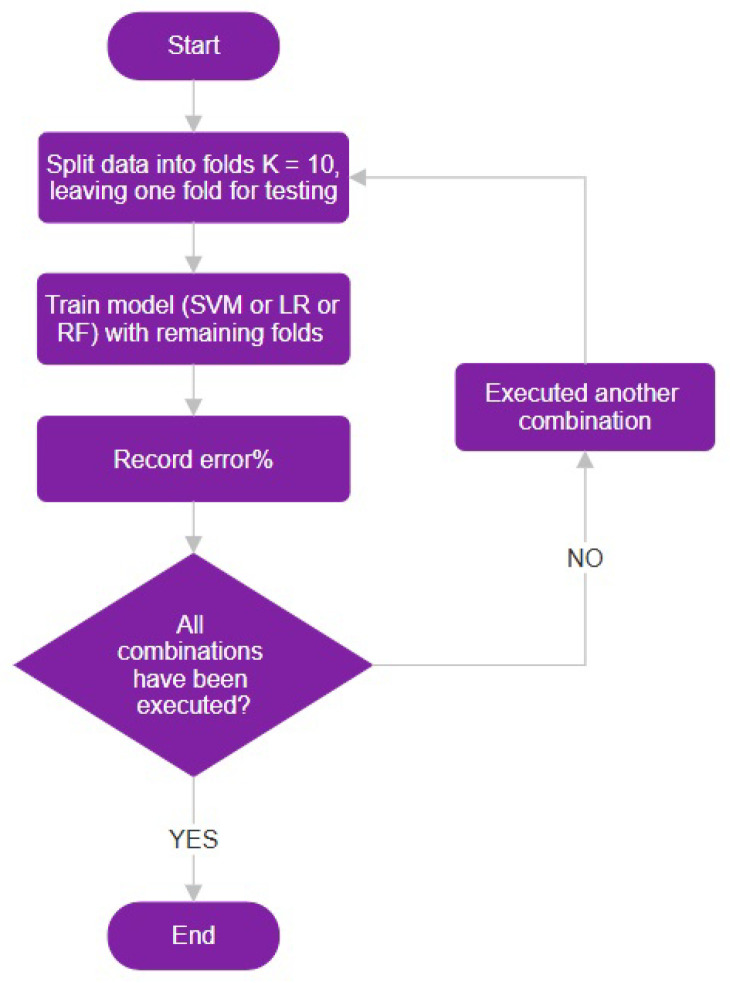
Illustration of the 10-fold cross-validation process, showing the division of the dataset into 10 equal-sized folds for model evaluation.

For the K = 10 cross-validation, we divided the dataset into 10 equal-sized folds such that each fold serves as the validation set once while the remaining folds serve as the training set (see Figure 12).

**Figure 13:**
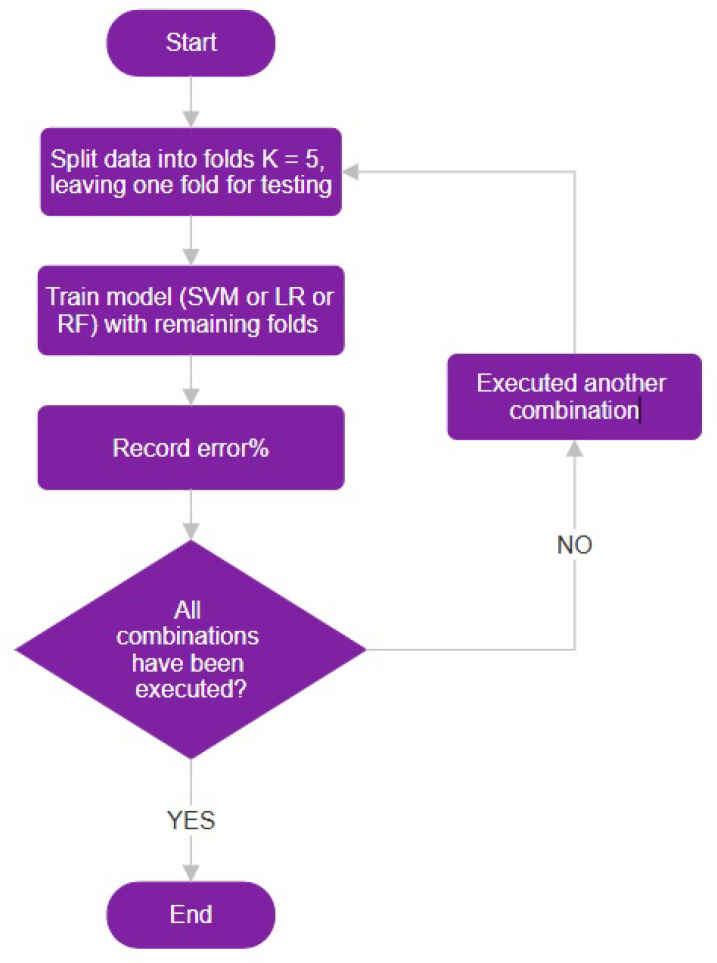
Illustration of the 5-fold cross-validation process, demonstrating the partitioning of the dataset into five subsets for model evaluation.

The K = 5 cross-validation was similar (see Figure 13).

**Figure 14:**
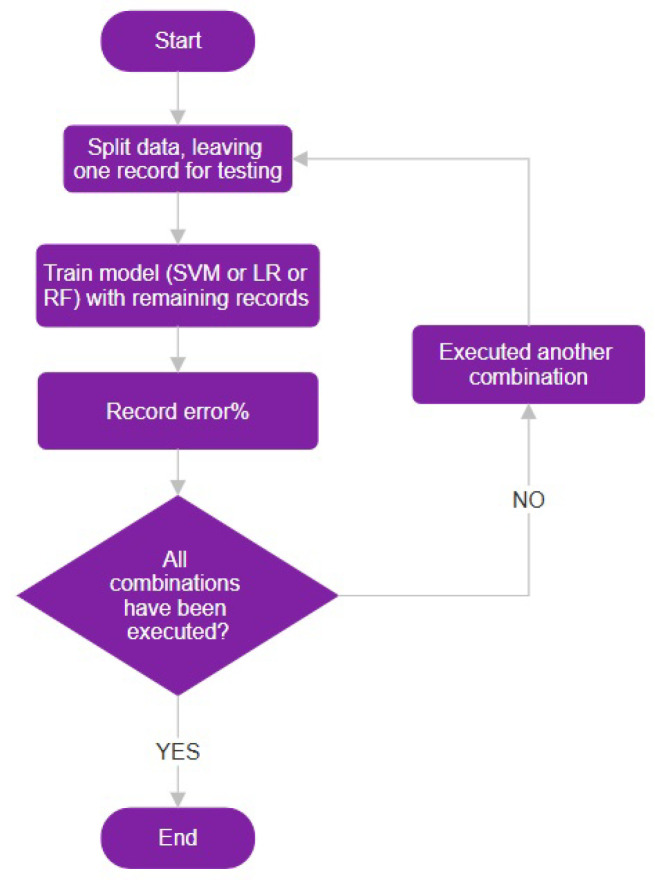
Illustration of the Leave-One-Out (LOO) cross-validation method, depicting the evaluation process where each individual data point serves as a separate validation set.

Finally, the Leave-One-Out method involved creating n folds, where n represents the total number of samples in the dataset, with each sample serving as a validation set once, while the rest of the data formed the training set (Figure 14).

We applied these techniques to Support Vector Machine (SVM), Random Forest, and Logistic Regression models. First, we used two methods to train our models: training models without feature selection and training models with mRMR feature selection and Mutual Information methods. This process culminated in the production of 27 distinct models. Additionally, we trained each model with and without outliers due to their strong influence on the model results.

We will provide a detailed analysis of the results obtained from each model, including SVM, LR, and RF, focusing on their performance metrics and implications.

#### 5.2.1. Support Vector Machines

**Figure 15:**
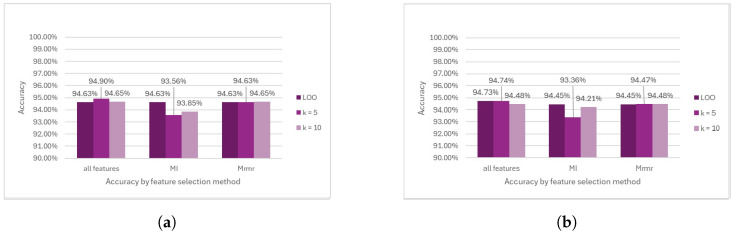
Comparison of SVM model performance. (**a**) Accuracy prior to outlier removal. (**b**) Accuracy following outlier removal.

SVM showed great performance before removing the outliers and achieved the highest accuracy among all the methods. Specifically, when no feature selection method was applied, in conjunction with K =5 during cross-validation, the SVM achieved an accuracy of 94.90%. Conversely, the lowest accuracy of 93.56% was observed when selecting MI features using K = 5 in the cross-validation process (see Figure 15a). However, after removing the outliers, the results derived from SVM remained largely consistent with those obtained before removing the outliers (see Figure 15b).

#### 5.2.2. Logistic Regression

**Figure 16:**
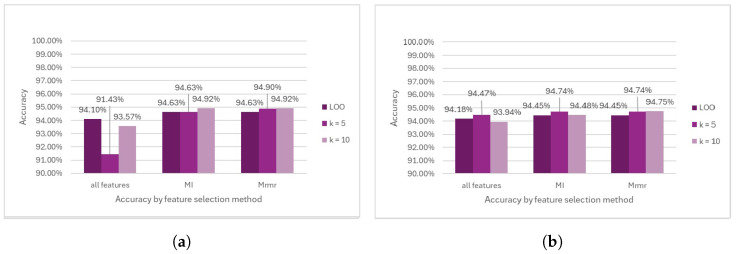
Comparison of LR model performance. (**a**) Accuracy prior to outlier removal. (**b**) Accuracy following outlier removal.

The results of Logistic Regression before removing the outliers showed remarkable performance, with the highest accuracy of 94.92% achieved by selecting the mRMR feature combined with K = 10 in the cross-validation procedure. In contrast, the lowest accuracy of 91.43% was recorded when no feature selection method was applied, in conjunction with K = 5 during cross-validation (see Figure 16a). After outlier removal, the Logistic Regression results showed no significant changes from the results before outlier removal (see Figure 16b).

#### 5.2.3. Random Forest

**Figure 17:**
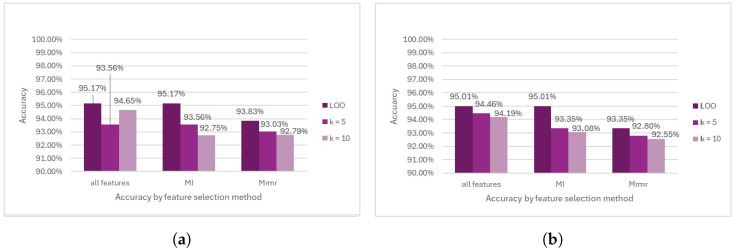
Comparison of Random Forest model performance. (**a**) Accuracy prior to outlier removal. (**b**) Accuracy following outlier removal.

As for RF before removing the outliers, the results showed a difference in performance from those obtained from the Logistic Regression analysis, where the highest accuracy was also observed at 95.17% by using all the features combined with Leave-One-Out cross-validation. Conversely, the lowest accuracy of 92.75% coincided with those scenarios where the MI was close to K = 10 during the cross-validation (see Figure 17a). For RF with outlier removal, the results showed no changes from the results before the outlier removal (see Figure 17b).

## 6. Discussion

Conducting this experiment has sparked inquiries into the sensitivity of the 70–30 split methodology to outliers. While the initial performance was commendable, the removal of the outliers notably improved the performance, contrasting with the minor impact observed in the cross-validation results post-outlier removal. The following tables show all the results we have achieved.

In Table 7, the results obtained from the hold-out method are displayed, including the accuracy and error rates with and without outliers.

As for the cross-validation method, the results we obtained from the cross-validation models did not show significant modifications after applying the outlier removal. In most cases, the accuracy decreased rather than improved after removing the outliers. In Table 8 below, the results obtained from the cross-fold method are displayed, including the accuracy and error rates with and without outliers.

Despite the improved outcomes observed following the outlier removal within the hold-out methodology, we think keeping the outliers within the dataset is better. We firmly believe that these outliers hold pivotal significance in accurately diagnosing various conditions that the model may encounter, especially if it is applied in real-world settings, thus underscoring their indispensable role in model robustness and generalization.

Three feature selection methods were used; we can see that the selected features play a crucial role in assessing cognitive impairment, brain atrophy, and cognitive reserve, making them clinically relevant. Integrating these features into clinical decision-making can help clinicians in personalizing patient care, enabling early intervention for those at higher risk.

All three models were trained using the same random seed value to ensure consistency across the experiments. This approach enables a fair comparison of model performance by minimizing the variability introduced by random sampling. In clinical use, Logistic Regression, with its simplicity and easily interpretable coefficients, can offer clear insights for clinicians. Random Forest, while slightly more complex, provides feature importance rankings that can help in identifying the key clinical factors. SVM, although highly accurate, is less interpretable, which may limit its standalone use in clinical settings.

## 7. Conclusions and Future Work

In this study, we experimented with several feature selection methods and classifiers to predict Alzheimer’s disease (AD) based on the OASIS-2 [15] dataset, which consists of a longitudinal cohort of 150 people aged 60 to 96 years. After analyzing the OASIS-2 dataset, our results show that mRMR and the correlation coefficient have the same effect on our classifiers. Mutual Information showed slightly different results from the other two approaches.

According to these results, mRMR and Mutual Information are effective methods for feature selection in predicting Alzheimer’s disease using the OASIS-2 dataset. In addition, we used three distinct classification methods: SVM, Logistic Regression, and Random Forest. Each model was trained without using feature selection and with using the mRMR and Mutual Information feature selection methodologies.

Our results showed that Logistic Regression produced similar results to SVM despite their different methodologies and methods, which essentially confirms the reliability of both models in achieving high accuracy, while the Random Forest model showed slightly lower performance compared to the other models. We also applied two approaches to train the models: a hold-out approach with 70% of the data allocated to training and the remaining portion allocated to testing, and a k-fold cross-validation approach.

In k-fold validation, we used different configurations of k, specifically using k = 10, k = 5, and Leave-One-Out (LOO) strategies. We applied these methods with and without removing outliers. According to our results, the 70–30 split methodology showed sensitivity to outliers. Although it showed commendable results before removing the outliers, removing the outliers significantly improved the performance. In contrast, we observed little impact on the validation results after removing the outliers.

There are several key factors that influenced our work throughout this research. First, feature selection is a major factor that affects the efficiency of the model and the overall quality of the results. In the future, we will focus on applying more advanced feature selection techniques, such as redundant feature elimination (RFE). Another factor is that we relied on the OASIS-2 dataset alone to train the model. In future work, we will focus on training our model on a larger and more comprehensive dataset, Also, in our future work, we will focus on using other types of machine learning algorithms than the ones we used to find out which model performs best, which ultimately improves the accuracy of the final results.

This research aimed to develop models for the early detection of Alzheimer’s disease, contributing to advancements in healthcare and education. By improving the accuracy and effectiveness of these models, we hope to ensure their accessibility for those individuals and families affected by Alzheimer’s disease. Our findings could lead to significant advancements in diagnostic tools, ultimately improving human well-being and quality of life.

Ultimately, we hope that our findings will have significant implications for the development of more accurate diagnostic tools for the early detection of Alzheimer’s disease.

## Figures and Tables

**Table 1:** Comparison of related ML models for dementia prediction in state-of-the-art approaches.

Study	Dataset	Feature Selection Technique	Classifier(s)	Accuracy
Saratxaga et al. [16]	OASIS-2	None	BrainNet2D (CLR triangular)	92%
			BrainNet3D (Batch Norm.)	84%
			ResNet18 (CLR triangular)	93%
Battineni et al. [17]	OASIS-2	Feature Exclusion	SVM	68.75%
Kavitha et al. [18]	OASIS-2	Correlation, Info gain, Chi-Square	Decision Tree	80.46%
			SVM	86.92%
			Random Forest	81.67%
Baglat et al. [19]	OASIS-2	None	Logistic Regression (Imputation)	78.9%
			Logistic Regression (Dropping)	75%
			Decision Tree	81.5%
			Random Forest	86.8%
			SVM	81.5%
Basheer et al. [20]	OASIS-2	None	M-CapNet	92.39%
Dhakal et al. [21]	OASIS-2	None	Decision Tree	89.5%
			K-Nearest Neighbor	95.1%
			Logistic Regression	94.35%
			Random Forest	94.35%
			SVM	96.77%
		LASSO	Decision Tree	89.5%
			K-Nearest Neighbor	95.1%
			Logistic Regression	94.35%
			Random Forest	94.35%
			SVM	94.35%
		Chi-Square	Decision Tree	91.93%
			K-Nearest Neighbor	94.35%
			Logistic Regression	94.35%
			Random Forest	94.35%
			SVM	94.35%

**Table 2:** Advantages and disadvantages of related ML models for dementia prediction in state-of-the-art approaches.

Study	Advantages	Disadvantages
Saratxaga et al. [16]	High accuracy (up to 93%) using advanced deep learning models like BrainNet2D and ResNet18. Applied optimization techniques such as CLR and Batch Normalization.	No feature selection used, which may include irrelevant data. Requires substantial computational resources.
Battineni et al. [17]	Simple implementation using SVM with moderate accuracy (68.75%).	Lower accuracy compared to other models, limiting effectiveness. Feature exclusion may overlook important predictors.
Kavitha et al. [18]	Good accuracy (up to 86.92%) with Random Forest and effective feature selection methods, such as correlation and chi-square.	Random Forest requires more computational power, and Decision Trees can overfit.
Baglat et al. [19]	Evaluated several classifiers, with Random Forest achieving 86.8% accuracy. Handled missing values through imputation.	Logistic Regression underperformed (75–78.9%), and inconsistent performance across classifiers makes it harder to choose the best model.
Basheer et al. [20]	Achieved high accuracy (92.39%) with M-CapNet, a specialized neural network.	No feature selection used, which may include unnecessary data.
Dhakal et al. [21]	Very high accuracy (up to 96.77%) using SVM and multiple classifiers like KNN, Random Forest, and Logistic Regression.	No specific feature selection was mentioned for SVM, and models could potentially overfit.

**Table 3:** Description of key features in the dataset.

Feature	Description
Group	Class label (Demented, Non-demented)
M/F	Male/Female
Age	Ranges from 60 to 96
EDUC	Years of Education
SES	Socioeconomic Status (1–5)
MMSE (Mini Mental State Examination)	A cognitive screening tool that provides a brief objective measure of cognitive function.
CDR (Clinical Dementia Rating)	A numeric scale used to quantify the severity of symptoms of dementia, including cognitive, functional, and social domains in the overall staging.
eTIV (Estimated total intracranial volume)	Volume of the cranial cavity.
nWBV (Normalized Whole Brain Volume)	Percentage of the intracranial cavity occupied by the brain.
ASF (Atlas Scaling Factor)	Volume-scaling factor required to match each individual to the atlas target.

**Table 4:** Group descriptions.

	Demented	Non-Demented	Converted
Description	a label given to patients with Alzheimer’s disease	a label given to patients without Alzheimer’s disease	a label given to patients diagnosed first to not have Alzheimer’s disease but were converted to be patients with Alzheimer’s disease
Count	146	190	37

**Table 5:** Encoding for group and M/F.

Group	M/F
Demented = 1	Female = 1
Non-demented = 0	Male = 0

**Table 6:** Summary of feature selection results.

Feature Selection	Selected Features
mRMR	CDR, MMSE, nWBV, EDUC, M/F
Correlation Coefficient	CDR, MMSE, nWBV, EDUC, M/F
Mutual Information (MI)	CDR, MMSE, EDUC, cTIV, ASF

**Table 7:** Results obtained from the hold-out method, showing accuracy and error rates with and without outliers.

Feature Selection	With Outliers						Without Outliers					
	SVM		Logistic Regression		Random Forest		SVM		Logistic Regression		Random Forest	
	Acc.	Error	Acc.	Error	Acc.	Error	Acc.	Error	Acc.	Error	Acc.	Error
**mRMR**	97.32%	2.68%	97.32%	2.68%	95.53%	4.47%	98.16%	1.84%	99.08%	0.92%	96.33%	3.67%
**MI**	97.32%	2.68%	97.32%	2.68%	95.53%	4.47%	98.16%	1.84%	98.16%	1.84%	98.16%	1.84%
**All Features**	96.42%	3.58%	97.32%	2.68%	95.53%	4.47%	98.16%	1.84%	98.16%	1.84%	98.16%	1.84%

**Table 8:** Results obtained from the k-fold cross-validation method, showing accuracy and error rates with and without outliers.

	With Outliers						Without Outliers					
	SVM		Logistic Regression		Random Forest		SVM		Logistic Regression		Random Forest	
	Accuracy/Error Rate	Std Dev	Accuracy/Error Rate	Std Dev	Accuracy/Error Rate	Std Dev	Accuracy/Error Rate	Std Dev	Accuracy/Error Rate	Std Dev	Accuracy/Error Rate	Std Dev
mRMR k = 10	94.65%/5.35%	0.039	94.92%/5.08%	0.030	92.79%/7.21%	0.055	94.48%/5.52%	0.040	94.75%/5.25%	0.037	92.55%/7.45%	0.057
mRMR k = 5	94.63%/5.37%	0.028	94.90%/5.10%	0.020	93.03%/6.97%	0.021	94.47%/5.53%	0.028	94.74%/5.26%	0.026	92.80%/7.20%	0.021
mRMR LOO	94.63%/5.37%	0.225	94.63%/5.37%	0.220	93.83%/6.17%	0.240	94.45%/5.55%	0.228	94.45%/5.55%	0.228	93.35%/6.65%	0.249
MI k = 10	93.85%/6.15%	0.050	94.65%/5.35%	0.030	92.75%/7.25%	0.062	94.21%/5.79%	0.050	94.48%/5.52%	0.040	93.08%/6.92%	0.063
MI k = 5	93.56%/6.44%	0.030	94.63%/5.37%	0.020	93.56%/6.44%	0.042	93.36%/6.64%	0.030	94.47%/5.53%	0.026	93.35%/6.65%	0.052
MI LOO	94.63%/5.37%	0.220	94.63%/5.37%	0.220	95.17%/4.83%	0.214	94.45%/5.55%	0.220	94.45%/5.55%	0.220	95.01%/4.99%	0.217
All features k = 10	94.65%/5.35%	0.033	93.57%/6.43%	0.060	94.65%/5.35%	0.033	94.48%/5.52%	0.045	94.48%/5.52%	0.052	94.19%/5.81%	0.035
All features k = 5	94.90%/5.10%	0.033	91.43%/8.57%	0.070	93.56%/6.44%	0.033	94.74%/5.26%	0.026	94.47%/5.53%	0.028	94.46%/5.54%	0.024

## Data Availability

The original data presented in the study are openly available at Washington University School of Medicine in St. Louis at https://sites.wustl.edu/oasisbrains/home/oasis-2/ (accessed on 20 January 2024).
